# Progress in Preparation of ZrB_2_ Nanopowders Based on Traditional Solid-State Synthesis

**DOI:** 10.3390/nano11092345

**Published:** 2021-09-09

**Authors:** Liuyang Bai, Yuge Ouyang, Fangli Yuan

**Affiliations:** 1 College of Energy Engineering, Huanghuai University, Zhumadian 463800, China; 2 College of Chemistry and Materials Engineering, Beijing Technology and Business University, Beijing 100048, China; ouyangyuge@btbu.edu.cn; 3 State Key Laboratory of Multiphase Complex Systems, Institute of Process Engineering, Chinese Academy of Sciences, Beijing 100190, China; flyuan@ipe.ac.cn

**Keywords:** ultra-high temperature ceramics, ZrB_2_ nanopowders, solid-state synthesis, self-propagating high-temperature synthesis, solution-derived precursors, plasma technology

## Abstract

ZrB_2_ is of particular interest among ultra-high temperature ceramics because it exhibits excellent thermal resistance at high temperature, as well as chemical stability, high hardness, low cost, and good electrical and thermal conductivity, which meet the requirements of high-temperature components of hyper-sonic aircraft in extreme environments. As raw materials and basic units of ultra-high temperature ceramics and their composites, ZrB_2_ powders provide an important way for researchers to improve material properties and explore new properties by way of synthesis design and innovation. In recent years, the development of ZrB_2_ powders’ synthesis method has broken through the classification of traditional solid-phase method, liquid-phase method, and gas-phase method, and there is a trend of integration of them. The present review covers the most important methods used in ZrB_2_ nanopowder synthesis, focusing on the solid-phase synthesis and its improved process, including modified self-propagating high-temperature synthesis, solution-derived precursor method, and plasma-enhanced exothermic reaction. Specific examples and strategies in synthesis of ZrB_2_ nano powders are introduced, followed by challenges and the perspectives on future directions. The integration of various synthesis methods, the combination of different material components, and the connection between synthesis and its subsequent application process is the trend of development in the future.

## 1. Introduction

Ultra-high temperature ceramics (UHTCs) mainly include refractory borides, carbides, and nitrides of some transition metals, such as ZrB_2_, HfB_2_, ZrC, and TaC, the melting points of which are usually above 3000 °C [1,2,3,4,5,6,7]. UHTCs and their composites have attracted great attention in the past two decades as potential heat-resistance candidates used in hyper-sonic aircraft and high-performance aircrafts [8,9,10,11,12,13,14,15]. Among these UHTCs, borides are considered superior due to their combination of excellent properties, including thermal shock resistance, creep resistance, and thermal conductivity [16,17,18,19]. Among all the borides, ZrB_2_ and HfB_2_ exhibit best oxidation resistance at high temperatures, as well as good electrical and thermal conductivity, chemical stability, and high hardness [20,21,22,23,24]. ZrB_2_ and HfB_2_ can realize long-time non-ablation in an oxidizing environment above 2000 °C. Furthermore, between these two diborides, ZrB_2_ has a relatively lower density and lower cost than HfB_2_, so it is preferred over HfB_2_ and mostly studied as industrially appealing ultra-high temperature ceramic powders [25].

ZrB_2_ powders not only act as the basic unit of UHTCs and their composites, but also provide an important way for researchers to improve material properties and explore new properties by way of synthesis design and innovation. To reach the extreme state of UHTCs, it is often necessary to change the original properties of materials, and the most effective methods to change the properties include particle refinement and recombination in the ultra-fine state [26]. Therefore, powder technology, especially powder synthesis technology, plays an important role in the field of UHTCs. As a matter of fact, in addition to powder synthesis, particle size and gradation control, modification, and assembly are also involved in the preparation of UHTCs and composites. Guo J. K. has pointed out that understanding physical and chemical problems in powder preparation, thermodynamics, and kinetics in the sintering process and the relationship between material microstructure and their properties is the basic guarantee for obtaining material reliability and stability [27,28].

Powders with high purity, ultra-fine, and uniform particle size are the basic raw materials for the preparation of advanced performance UHTCs and their composites [29,30]. It has been demonstrated that when the particle size decreases into the nanoscale, the ZrB_2_ powders exhibit an excellent sintering ability and facilitate the formation of nano-grained materials and C–C composites with improved mechanical properties. Therefore, different methods have been established for the synthesis of ZrB_2_ nanopowders [31,32,33,34,35,36,37]. Amongst them, solid-state synthesis, including the borothermic reduction method, carbothermic reduction method, and metallothermic reduction method, is mostly used because the process is simple and the raw materials are cheap and easily available. However, the solid-phase reaction between raw material components is difficult to complete. One cannot obtain high-purity uniform nanopowders directly and crushing is always necessary after high-temperature synthesis, which consumes a lot of extra energy and may adversely affect purity [38]. During the solid-state synthesis, especially when SHS is involved, the formation of coarse particles is unavoidable as a result of high temperature sintering [39].

In recent years, the development of ceramic powder synthesis methods has broken through the traditional classification of a solid-phase method, a liquid-phase method, and a gas-phase method, and there is a trend of integration of various methods, such as semichemical methods in which the precursors for solid-state synthesis are prepared using aqueous solution instead of solid raw materials to improve reaction activity, and the low-temperature combustion synthesis method combining the spray pyrolysis method of both liquid- and gas-phase methods [40,41,42,43,44].

There are many similar studies on the synthesis of ZrB_2_ nanopowders and significant progress has been made almost every year. The present review covers the most important methods used in ZrB_2_ nanopowder synthesis, focusing on the solid-phase synthesis and its improved process. Specific examples and strategies in the synthesis of ZrB_2_ nanopowders are introduced, followed by the challenges in ZrB_2_ nanopowder synthesis and the perspectives on future directions.

## 2. Solid-State Synthesis

Solid-state synthesis plays a unique important role in the preparation of ZrB_2_ powders. Generally, ceramic synthesis can be divided into gas-phase, liquid-phase, and solid-phase methods according to the state of reactants. However, boron-containing gases mainly include toxic boron trichloride and spontaneously combustible borane, the safety of which when used in large quantities is difficult to guarantee. Therefore, the solid-phase process has almost become the inevitable choice of ZrB_2_ powders.

Solid-state synthesis mainly includes direct reaction between elemental boron and zirconium powders, carbothermal reduction reaction, and magnesiothermic reduction reaction. In a direct reaction method, ZrB_2_ powders are obtained by completing the following reaction [45,46,47]:

Zr + 2B → ZrB_2_ (1)

The advantage of this method is that the purity of the powders is relatively high. However, the raw materials are expensive and the metallic zirconium is difficult to preserve and transport. The products are of large particle size and low sintering activity, which is not conducive to post-processing and final application. Therefore, the direct reaction method is difficult to realize in industrial production and also rarely appears in the latest literature [48,49].

In the carbothermal reduction reaction and magnesiothermic reduction reaction, zirconia (ZrO_2_) is applied as one of the raw materials to provide Zr for the synthesis of ZrB_2_, and carbon or magnesium are introduced into the reaction system to reduce ZrO_2_ to Zr. The reduction reactions are as follows:

ZrO_2_ + 2C → Zr + 2CO(g) (2)

ZrO_2_ + 2Mg → Zr + 2MgO (3)

These two reactions are exothermic, and the heat of the reactions promotes the continuous progress of the reaction in turn, which is the reason they are named carbothermal and magnesiothermic reduction reactions. When the heat of a reaction is sufficient, it will successively trigger the adjacent raw material layer to react and subsequently release more heat, so that the reaction automatically propagates in the form of a combustion wave until completion without any other energy supply from outside after initiation. This process is called self-propagation high-temperature synthesis (SHS) [50,51,52]. Adiabatic temperature (T_ad_) is a temperature that the exothermic reaction system can reach in an insulated environment, which is an important parameter to predict a SHS process. Varma A. proposed a thermodynamic criterion for judging the maintenance of a SHS process according to the value of T_ad_: if T_ad_ > 1800 K, the SHS can continue; if T_ad_ ˂ 1500 K, the heat released by the reaction is not enough to make the combustion reaction continue; if 1500 K ˂ T_ad_ ˂ 1800 K, the system must be provided with additional energy from the outside to continue [53,54].

The common carbothermal reduction systems for synthesizing ZrB_2_ powders include ZrO_2_-B_4_C-C, ZrO_2_-B_2_O_3_-C, and ZrC-B_2_O_3_-C as below:

2ZrO_2_ + B_4_C + 3C → 2ZrB_2_ + 4CO(g) (4)

ZrO_2 +_ B_2_O_3_ + 5C → ZrB_2_ + 5CO(g) (5)

ZrC + B_2_O_3_ + 2C → ZrB_2_ + 3CO(g) (6)

The magnesiothermic reduction system of Mg-ZrO_2_-B_2_O_3_ is usually chosen for synthesizing ZrB_2_ powders as below:

ZrO_2_ + B_2_O_3_ + 5Mg → ZrB_2_ + 5MgO (7)

The calculated T_ad_ of Equation (7) is 3085 K, which is much higher than 1800 K, so the reaction system of Mg-ZrO_2_-B_2_O_3_ is a typical SHS system, which is the most-used technique for ZrB_2_ powders both in laboratory and industry [55,56,57].

## 3. Modified Self-Propagating High-Temperature Synthesis

In a general SHS process for the production of ZrB_2_ powders, there are mainly four steps. The first is to prepare green reactive mixture; the second to press the green mixture; the third is to sinter using the SHS process; and fourth is to post-treat the SHS products. There are two ways to initiate the SHS reaction. One way is to heat reactants in a small area and initiate the reaction from one point, and the exothermic combustion would consequently heat the surrounding reactants and self-sustained propagation throughout the whole area. The other way is that the entire body of the green reactive mixture is pre-heated uniformly in a high temperature environment until the reaction is initiated overall [52,58].

Though the reaction is initiated from a small area and self-propagated throughout the whole body in a classical SHS method, cold pressing of the green mixture into dense compacts is usually necessary to facilitate heat and mass transfer between reactants. However, pressed green compacts would lead to serious sintering and agglomeration of the products, which exist in the form of porous sintered blocks. Further milling of the sintered products and sieving of the powders to desired grades is needed.

**Figure 1:**
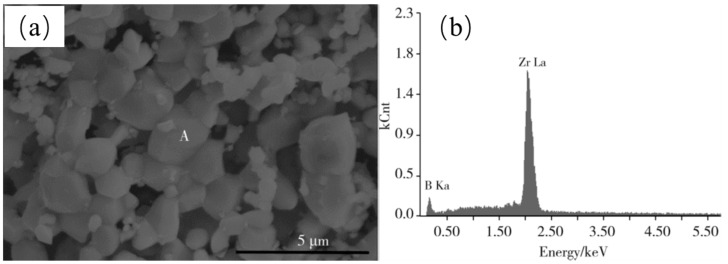
FESEM image of ZrB_2_ powders (**a**) and EDS of point A (**b**) [60].

**Figure 2:**
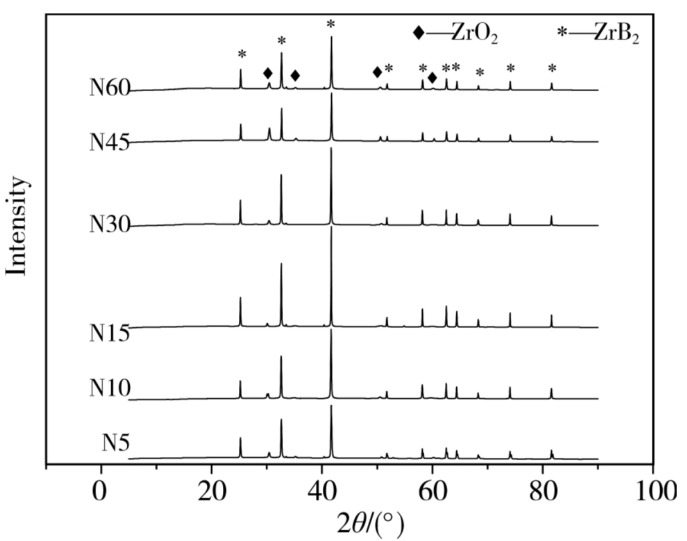
XRD patterns of ZrB_2_ powders prepared with different content of NaB_4_O_7_ [60].

In order to solve the problem mentioned above, different additives were introduced into the SHS system in order to adjust the combustion temperature and isolate ZrB_2_ particles from each other to get well-dispersed nanoparticles. Zhang W. studied the Mg-ZrO_2_-B_2_O_3_-MgCl_2_ and Mg-ZrO_2_-B_2_O_3_-NaB_4_O_7_ system and Zhang T. studied the Mg-ZrO_2_-B_2_O_3_-MgO system for the preparation of ZrB_2_ powders by SHS [59,60,61].Figure 1 shows FESEM images of the ZrB_2_ powders synthesized without any diluents and the EDS image of point A. Figure 2 shows XRD patterns of ZrB_2_ powders prepared with different content of NaB_4_O_7_.

Results showed that an optimum amount of MgCl_2_ and NaB_4_O_7_ could decrease the grain size of ZrB_2_ powders effectively, while there was also a significant improvement in ZrB_2_ purity. Particle size of ZrB_2_ powders decreased with the increase of MgO content and reached the lowest value of 0.41 μm when the content of MgO was 30%, and the specific surface area was the biggest at 20.02 m^2^/g. The purity of ZrB_2_ powders could be raised by increasing Mg and B_2_O_3_ content in the raw materials. When stoichiometric amounts of Mg, ZrO_2_, and B_2_O_3_ were mixed as reactants in which Mg is 30% excessive and B_2_O_3_ is 5% excessive, the purity of ZrB_2_ powders was the highest at 96.31% with the Zr content of 77.88%, the B content of 18.43%, and the O content of 1.28%.

Khanra A. K. synthesized ultrafine ZrB_2_ powders by SHS from a mixture of H_3_BO_3_, ZrO_2_, and Mg. The experiments were carried out within a tubular furnace and Ar flow was provided continuously into the reaction system. The crystallite sizes of ZrB_2_ powders calculated using Scherrer formula based on XRD characterization results are given in Table 1. The addition of NaCl to the green mixture as inert diluent decreased the combustion temperature and helped yield ultrafine ZrB_2_ powders [62]. NaCl melted and vaporized partially in the synthesis process and became coated on ZrB_2_ particles, inhibiting the nanograin from growing into big particles. NaCl addition also decreased the adiabatic temperature, which might also contribute to the reduction of the grain size.

The other approach for ZrB_2_ powder production is to use a non-pressed green mixture to avoid sintering and agglomeration at high temperature. Heat and mass transfer become difficult due to the porosity of non-compacted samples, and the reaction has difficulty in automatically propagating without any energy supply from outside after initiation. Therefore, the entire body of the sample needs to be placed in a high temperature environment and heated uniformly. Since the melting point of B_2_O_3_ is only 450 °C, the green mixture will become inhomogeneous due to B_2_O_3_ loss during the pre-heating process. The heating temperature of the furnace needs to be higher than the reaction temperature when ΔG < 0. Some researchers used B instead of B_2_O_3_ as a raw material to overcome B loss. Moreover, the fast heating technique provided another strategy to eliminate segregation and loss of low melting/boiling point raw materials. Field-assisted heating technologies represent an excellent experimental base for researchers engaged in the discovery of new materials, which opens up wide opportunities for modeling the processes of materials consolidation and synthesis while taking into account numerous physical phenomena [63].

We synthesized well-dispersed submicro ZrB_2_ powders using a Mg-ZrO_2_-B reaction system, in which Mg, ZrO_2_, and B were intimately mixed by ball milling to get the green mixture and packed directly in a graphite crucible without being pressed into a high-density compact material. A fast heating and high temperature furnace were designed to guarantee the heat and mass transfer between naturally packed raw materials and decrease Mg loss at the pre-heating and initial stages of the reaction. In this work, the low packing density of the green mixture and high heating temperature of the designed induction heating furnace were crucial for the formation of well-dispersed submicro powders [64]. The heating program had great effects on the purity and particle size of the synthesized ZrB_2_ powders.

**Figure 3:**
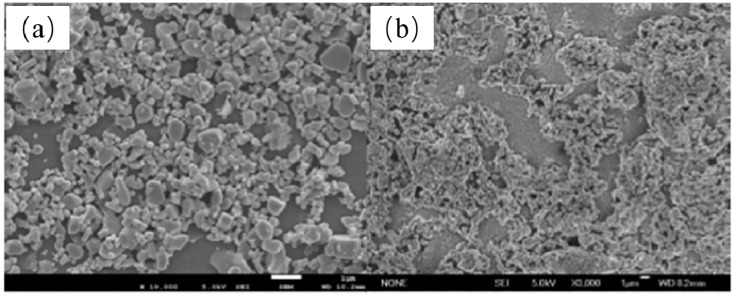
SEM picture of the sample obtained using green mixture with packing density of 0.66 g/cm^3^ (**a**) and 1.32 g/cm^3^ (**b**) [64].

It can be seen from Figure 3 that well-dispersed ZrB_2_ powders were obtained when the green mixture was naturally packed in the crucible, while aggregates appeared in a large area when the packing density increased from 0.66 g/cm^3^ (a, naturally packed) to 1.32 g/cm^3^ (b, twice that of the naturally packed). When the compact density of the green mixture is low, specific heat of the samples also become slow because pores between raw materials block heat transfer and mass transfer in the solid-state reaction. We designed a fast-heating furnace of 1600 °C to provide enough energy to make up for the problem of insufficient reaction caused by insufficient heat transfer. It can be seen that the high compact density of the green mixture is an unnecessary condition when the high temperature furnace is applied.

**Figure 4:**
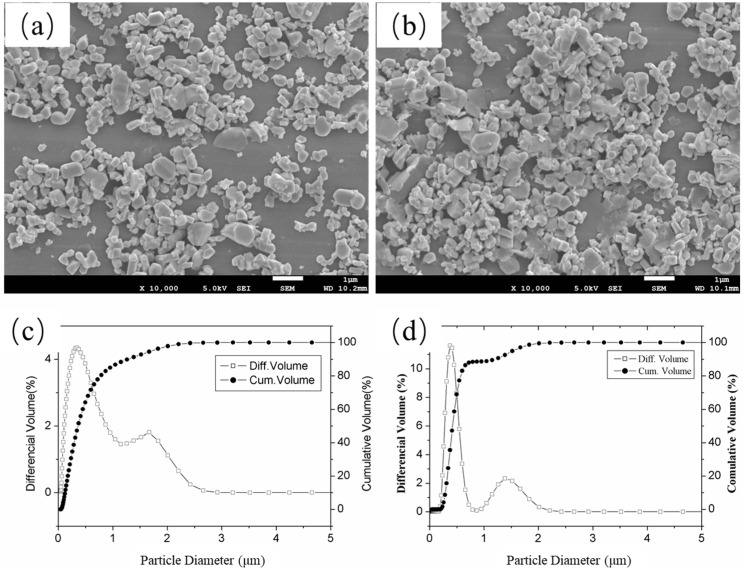
SEM images and particle size distribution of samples obtained following the primary synthesis step (**a**,**c**) and the second synthesis step (**b**,**d**) [65].

We further developed the method for the synthesis of ZrB_2_ powders with low oxygen content by a two-step reduction route. ZrB_2_ powders were synthesized in the first step using Mg-ZrO_2_-B reaction system as before. Then, in the second step, ZrB_2_ powders obtained from the first step were mixed with Mg at a mass ratio of ZrB_2_ to Mg being 10:1 and subjected to heating in the designed furnace again [65]. Figure 4 shows the FESEM images and corresponding particle size distribution of the samples obtained following the first step (a and c) and the second step (b and d). It can be seen that oxygen content decreased after another second synthesis step, while minor change could be observed in the particle size and dispersion of ZrB_2_ powders.

**Figure 5:**
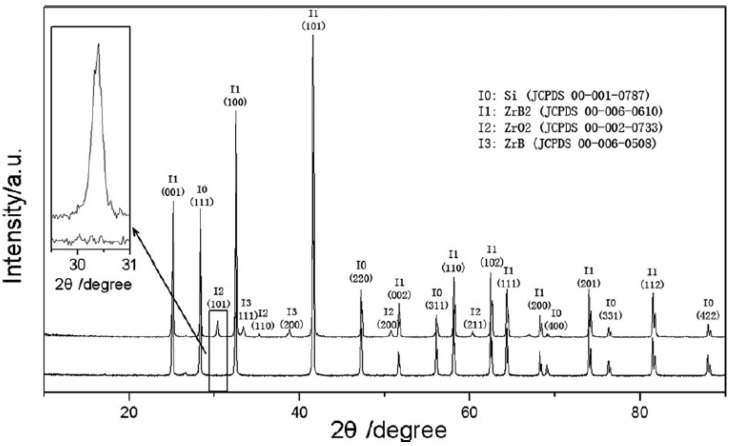
XRD patterns of samples mixed with the guide Si plate [65].

A new method for oxygen content calculated based on XRD results for ZrB_2_ characterization was proposed in this work. Figure 5 shows XRD patterns of samples mixed with the guide Si plate. It was discovered that the lattice constants determined according to XRD patterns were higher than their theoretical values calculated based on the First Principles. The O atom existing as interstitial impurity in the ZrB_2_ crystal lattice might contribute to the lattice constant changes.

## 4. Solution-Derived Carbothermal Synthesis

In a typical carbothermal synthesis of ZrB_2_ powders, carbon is used as a reducing agent and the reaction is as Equation (5). Raw materials are cheap and the technological process is simple, which makes it easy for industrial amplification. However, it was calculated that the reaction temperature is 1509 °C at atmospheric pressure according to the criterion that ΔG < 0. In contrast, ΔG for the magnesiothermic synthesis Equation (7) is always below zero from room temperature to 1400 K. It can be seen that carbothermal synthesis can only be achieved when the temperature is high enough. As the results of experiments, when the synthesis temperature was 1400 °C, the diffraction peaks of ZrB_2_ in the XRD patterns started to become obvious, but there were still strong diffraction peaks of raw materials ZrO_2_. When the reaction temperature increased to 1550 °C (>1509 °C), the diffraction peaks of raw materials disappeared, but the diffraction peaks of ZrC showed up instead [55].

The direct carbothermal method for synthesizing ZrB_2_ powders still has disadvantages, such as high temperature of reaction, low purity, and sintering activity of the products. Therefore, how to lower the reaction temperature is a critical task for the successful synthesis of ZrB_2_ powders by carbothermal reduction.

Synthesis in vacuum is a commonly used improved process. The change of Gibbs’s free energy can be used to determine whether the reaction can happen from thermodynamics. When ∆G < 0, the reaction is spontaneous; when ∆G > 0, the reaction is not spontaneous. The value of Gibbs free energy change in a non-standard state can be calculated based on that of the standard state according to Equation (8):

∆G = ∆G^0^ + RTlnK_p_ (8)

When the synthesis of ZrB_2_ powders is carried out under vacuum, the air pressure in the furnace is supposed to remain below 11 Pa; it can be considered that the partial pressure in the furnace is 11 pa. If one chooses 11 Pa as the partial pressure of CO in the furnace, the minimum reaction temperature (∆G = 0) of Equation (5) is 938 °C, which is much lower than 1509 °C under the standard state [55].

**Figure 6:**
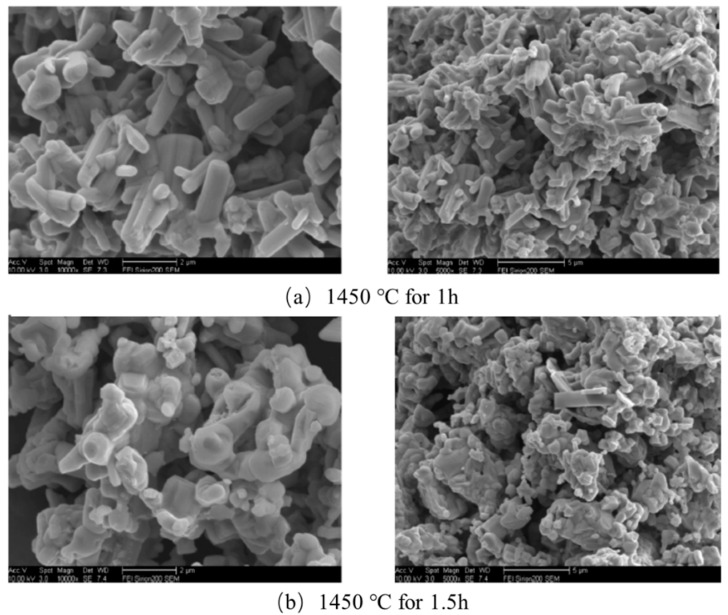
FESEM images of ZrB_2_ powders synthesized at 1450 °C for different holding times ((**a**) 1 h; (**b**) 1.5 h) [66].

Lv G. prepared ZrB_2_ powders by a carbon reduction method with zirconia, activated carbon, boron carbide, and boron oxide as main raw materials under vacuum, and obtained ZrB_2_ powders of high purity, small particle size, and low cost [66]. Figure 6 shows the FESEM images of ZrB_2_ powders synthesized at 1450 °C for different holding times.

**Scheme 1:**
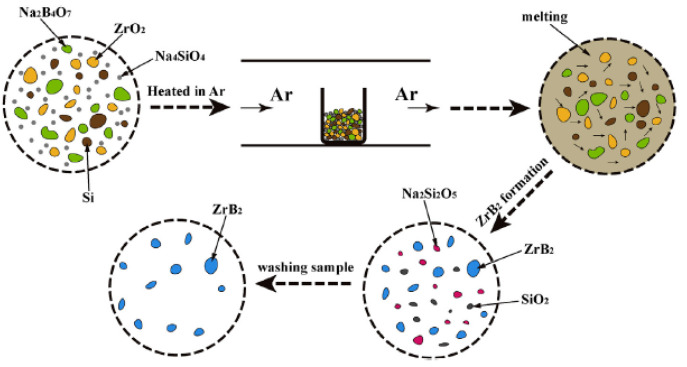
Schematic diagram for the preparation of ZrB_2_ [68].

Li M. reported a molten-salt method for the synthesis of ZrB_2_ powders [67]. Scheme 1 shows a schematic diagram for the preparation of ZrB_2_ powders. The participation of Na_4_SiO_4_ and Na_2_B_4_O_7_ provided a liquid surrounding that could dissolve reactant species and promoted rapid diffusion between them. Molten salt helped shorten the average diffusion distance and lower the reaction temperature.

Solution combustion synthesis (SCS) is a new approach for the production of ZrB_2_ powders. Initial reactants are solved in a homogeneous solution and form uniform precursors at the molecular scale. In a SCS process, a huge amount of gas would be generated and make the solid product expanded and porous, which is critical for SCS to synthesize nano-ZrB_2_ powders [68,69].

**Scheme 2:**
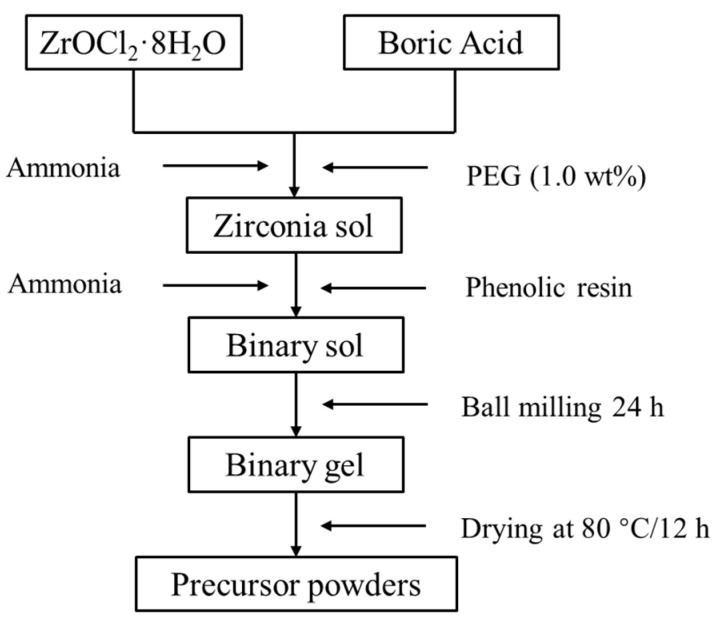
Synthesis flowchart for precursors [70].

**Figure 7:**
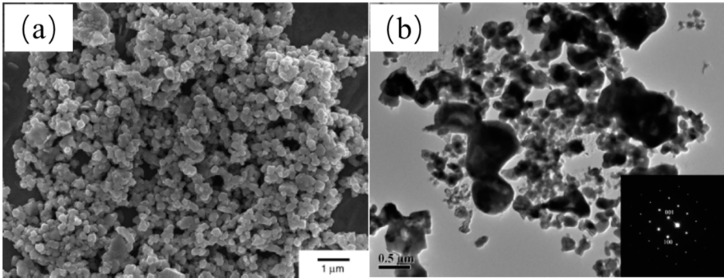
FESEM (**a**) and TEM (**b**) images of the synthesized ZrB_2_ powders [70].

Yan Y. synthesized ultra-fine ZrB_2_ powders using an inorganic–organic hybrid as a precursor with zirconium oxychloride as the source Zr, boric acid as the source of B, and phenolic resin as the source of C [70]. The carbothermal reaction temperature was at a relatively low level (1500 °C), and the average crystallite size of the obtained ZrB_2_ powders was small (200 nm). Scheme 2 shows the synthesis flowchart for precursors and Figure 7 shows the FESEM and TEM images of the synthesized ZrB_2_ powders.

Though ZrB_2_ powders can be synthesized from the precursors prepared by the sol-gel method, the precursors are usually of low effective concentration, poor stability, easy to settle and precipitate, and difficult to store. Therefore, the development of ultra-high temperature ceramics has extended from the sol-gel to the polymer precursor method [71,72,73]. One strategy is to synthesize the polymer with M–O as the main chain by chemical reaction, and then prepare the ultra-high temperature ceramic precursor with the compound containing C (phenolic acid) and B (boric acid). The other strategy is to synthesize a metal organic polymer with Zr–B chemical bonds in the molecules, which can be directly converted into ZrB_2_ ceramics by cracking.

Li R. used a sol-gel method to synthesize ZrB_2_ powders using Zr(OC_3_H_7_)_4_ (zirconium n-propoxide) as the source of Zr, H_3_BO_3_ (boric acid) as the source of B, C_12_H_22_O_11_ (sucrose) as the source of C, and acetylacetone as chemical modifier [74,75]. C_12_H_22_O_11_ could decompose completely to carbon, which might be accounted precisely according to stoichiometric ratio for the carbothermal reduction reaction. The process of precursor formation is as below:

Zr(OC_3_H_7_)_4_ + acac → Zr(acac)_2_ + C_3_H_7_OH (9)

≡Zr-OC_3_H_7_ + H_2_O → ≡Zr–OH + C_3_H_7_OH (10)

≡Zr-aca c+ H_2_O →≡ Zr–OH + aca (11)

≡Zr-OC_3_H_7_ + HO-Zr ≡ →≡ Zr–O–Zr ≡+ C_3_H_7_OH (12)

≡Zr-OH+HO-Zr ≡ → ≡ Zr–O–Zr ≡+ H_2_O (13)

Metal alkoxides can react with water spontaneously and form precipitation after continuous hydrolysis and condensation. In the literature, acetylacetone (acac) chelates with Zr (OC_3_H_7_)_4_ to form zirconium acetylacetonate, so that Zr(OC_3_H_7_)_4_ was modified to prevent its rapid hydrolysis. Yellow ZrO_2_ sol can be formed after the above series of reactions, and wet gel can be formed after mixing with H_3_BO_3_, C_12_H_22_O_11_, and AcOH. Finally, precursor can be obtained by the drying and grinding of the wet gel, and a single phase ZrB_2_ powder with the average grain size of 50 nm can be obtained after being calcined at 1550 °C.

Another process of precursor formation is the modification of Zr(OC_3_H_7_)_4_ with complexing agent AcOH to prevent its rapid hydrolysis [75,76,77]. The reactions are as below:

Zr(OC_3_H_7_)_4_ + 2AcOH → Zr(OAc)_2_(OC_3_H_7_)_2_ + 2C_3_H_7_OH (14)

C_3_H_7_O − Zr(OAc)_2_ − OC_3_H_7_ + HO−C_6_H_10_O_4_−(OH)_7_ → C_3_H_7_O − Zr(OAc)_2_-O-C_6_H_10_O_4_-(OH)_7_ + C_3_H_7_OH (15)

AcOH + C_3_H_7_OH → C_3_H_7_OAc + H_2_O (16)

Reaction (14) takes place when Zr(OC_3_H_7_)_4_ is added into AcOH, which leads to the formation of zirconium propoxide diacetate Zr(OAc)_2_(OC_3_H_7_)_2_ followed by a transesterification of alkoxy groups in Zr(OAc)_2_(OC_3_H_7_)_2_ with the saccharose OH groups according to Equation (15). The unreacted AcOH reacts with the solvent C_2_H_5_OH (which is also the product in reaction (14) and (15)) according to Equation (16). The product water reacts with Zr(OAc)_2_(OC_3_H_7_)_2_ spontaneously as Equation (17). Equation (18) occurs to generate oxo ligands by non-hydrolytic condensation and the elimination of an ester.

Zr(OAc)_2_(OC_3_H_7_)_2_ + 2H_2_O → Zr(OAc)_2_(OH)_2_ + 2C_3_H_7_OH (17)

Zr(OAc)_2_(OH)_2_ → (C_3_H_7_O)Zr-O-Zr(OAc) + C_3_H_7_OAc (18)

After formation of molecules that have hydroxy groups, subsequent condensation reactions take place resulting in the viscosity increase of the solution because of the formation of Zr-O-Zr and Zr-O-X-O-Zr bridges. When all the reactions are completed, a yellow colloid is obtained. The precursor powders are obtained by drying and grinding.

Li R. also used zirconium n-propoxide, acetic acid, boric acid, and xylitol as starting materials to synthesize ZrB_2_ nanoparticles by sol-gel method and subsequent carbothermal reduction reaction [78]. Xylitol played the roles as both the carbon source for the carbothermal reduction reaction and the chelating agent of boron in this synthesis system. The polyhydroxy reaction between xylitol and boric acid contributed to the formation of coordination compound.

**Figure 8:**
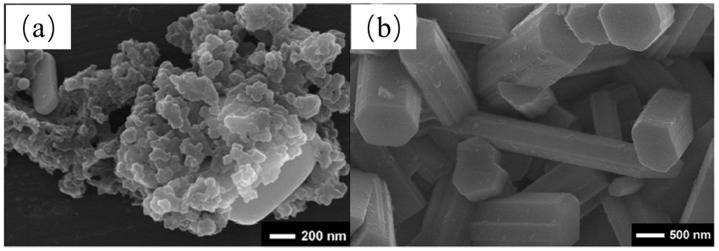
FESEM images of precursor derived from the nascent state gel calcined at (**a**) 1300 °C and (**b**) 1550 °C for 2 h, respectively. [79].

The gel aging time and calcination had great effects on the product morphology. When ZrB2 nanoparticles were obtained from nascent state gel calcined at 1450 °C with n(xylitol)/n(zirconium n-propoxide) of 1.4, the particles were quasi spherical in shape with an average diameter of about 50 nm, and there was an agglomeration to some extent. When ZrB2 nanoparticles calcined at 1550 °C for 2 h using the aged gel with molar ratios of B/Zr = 3.5 and C/Zr = 7, the particles are uniform and exhibit a long column shape with a length of 4–7 μm and the diameter is about 1 μm. Figure 8a,b displays the FESEM images of precursor derived from the nascent state gel calcined at 1300 °C and 1550 °C for 2 h, respectively. We can see that hexagonal-prism-like particles emerged calcined at 1550 °C instead of aggregates obtained at low 1300 °C [79].

**Scheme 3:**
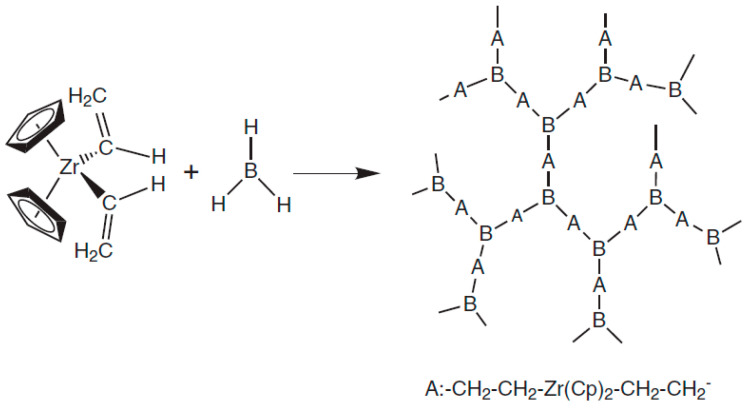
Synthesis of precursor PZCB [80].

Wang H. reported a polymer precursor-derived method for the synthesis of ZrC/ZrB_2_ composite powders [80]. The precursor polymer called PZCB that was prepared using Cp_2_Zr(CHCH_2_)_2_ and borane, providing elements B and Zr for subsequent carbothermal synthesis. The formation of precursor polymer is shown in Scheme 3.

## 5. Plasma-Enhanced Exothermic Reaction

**Figure 9:**
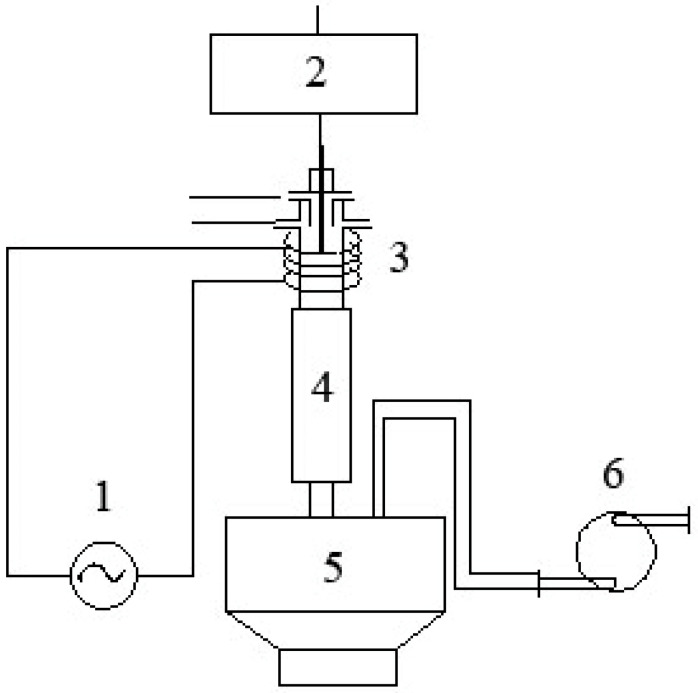
Layout of the RF thermal plasma processing system: (1) plasma power supply system, (2) entrained-flow powder feeder, (3) plasma torch, (4) cylindrical reactor and cooling chamber, (5) powder collector, (6) vacuum pump and exhaust system [90].

**Figure 10:**
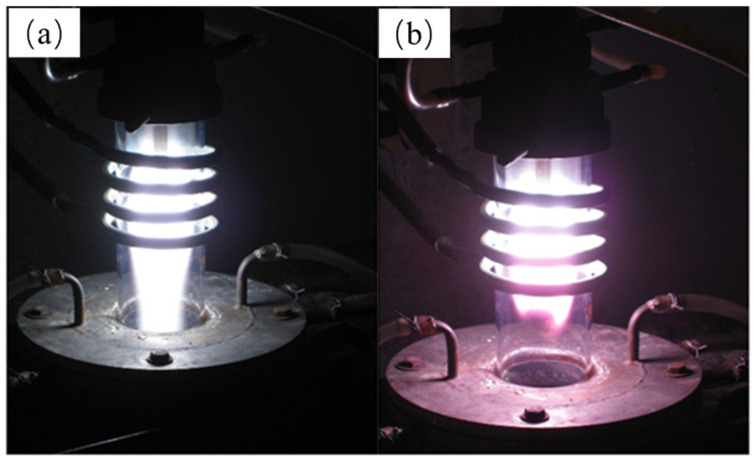
RF plasma jet ((**a**) Ar-plasma; (**b**) H-plasma) [90].

Thermal plasma synthesis is the most efficient technique for producing metal and ceramic nanopowders in continuous and scalable way [81,82,83,84,85,86,87]. In addition to the characteristics of high temperature, high energy density, and the fast cooling rate of a thermal plasma, radio frequency (RF) induction plasma has no electrode pollution and provides a controllable atmosphere [88,89]. Figure 9 shows the layout of the RF thermal plasma processing system and Figure 10 shows pictures of Ar-plasma and H-plasma jets [90].

**Scheme 4:**
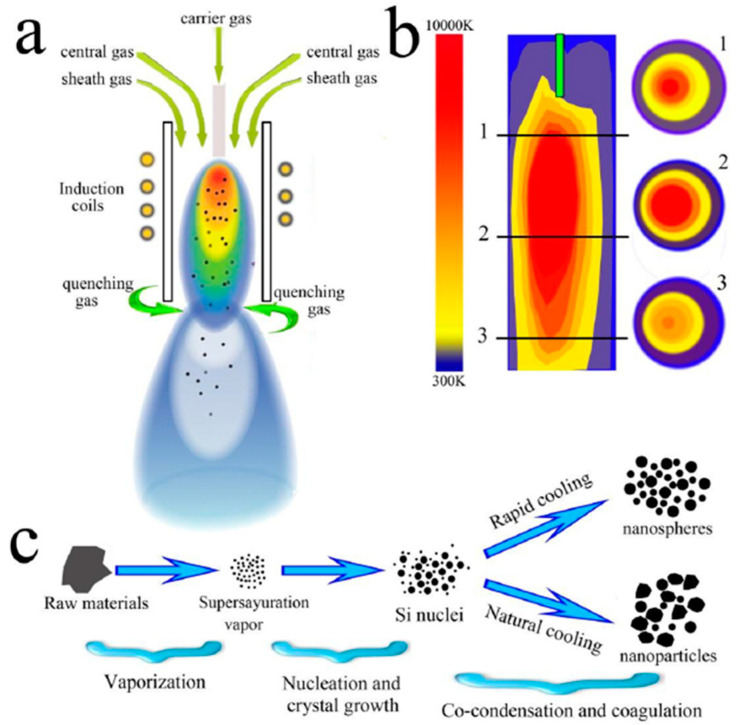
(**a**) sketch of the RF plasma reactor, (**b**) temperature thermofluid field in plasma arc and (**c**) schematic diagram of formation process of nanopowder in plasma [83].

Generally, plasma synthesis includes physical vapor deposition (PVD) and chemical vapor deposition (CVD). In a PVD process, coarse particles are injected into the plasma flame and gasified at high temperature and nanopowders are deposited after being rapidly cooled under large temperature gradient. Scheme 4 shows the diagram of the formation process of nanopowder in plasma. In a CVD process, more than two reactants are added into the plasma flame together, which are usually gasified at high temperature and react with each other. Ultrafine metal/oxide powders and various nanostructures can be obtained via this instantaneous enhanced reduction/oxidation reaction of active hydrogen or active oxygen provided by thermal plasma.

Great progress has also been made for the high temperature ceramic powders such as carbides and nitrides that require high synthesis temperature beyond conventional heating methods in recent years [91,92,93,94]. Generally, gases react directly in the plasma arc or low boiling point raw materials are added into the plasma arc to obtain ultra-fine powder through gasification reaction deposition. Almost all of these kinds of processes are characterized by using gases such as chloride and nitrogen/hydrocarbons or using solid organisms with low boiling point and easy decomposition as raw materials. A multiphase complex system in which more than one high boiling point raw materials participates in the reaction are generally considered not suitable for thermal plasma synthesis. Although the temperature and energy density in the thermal plasma arc area are very high, the gas flow rate in the reactor is very large so the residence time of raw materials in the plasma arc is limited, and the high boiling point raw materials do not easily realize volatilization and continuous gas phase reaction in a short time, which greatly limits its application fields.

Table 2 shows a comparison list between the characteristics of a plasma process and the properties of ZrB_2_ powders. They match perfectly with each other, which guides us to explore a most suitable way to prepare high-purity ZrB_2_ nano powders using plasma technique.

SHS is widely used in the synthesis of ultra-high temperature ceramic powders. Typically, a reaction is initiated from a small area and self-propagating throughout the whole body. In the section “Modified self-propagating high-temperature synthesis”, it was concluded that ZrB_2_ powders could also be produced using a non-pressed green mixture when the reaction system was placed in a high temperature environment and heated uniformly. Therefore, we further adjusted the traditional solid-phase self-propagating system. The green mixture was converted into fluidization by carrier gas and transported into the plasma flame with extremely high temperature. In order to adapt to the characteristics of rapid synthesis in plasma, ZrCl_4_, which has a low boiling point, was used as raw material instead of ZrO_2_ which has a high boiling point. The plasma synthesis process has been successfully applied to multiphase complex reaction systems with exothermic characteristics.

**Scheme 5:**
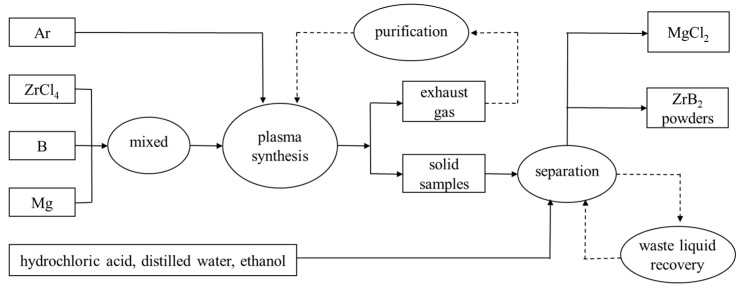
Process flow chart for plasma synthesis of ultrafine ZrB_2_ powders [96].

**Figure 11:**
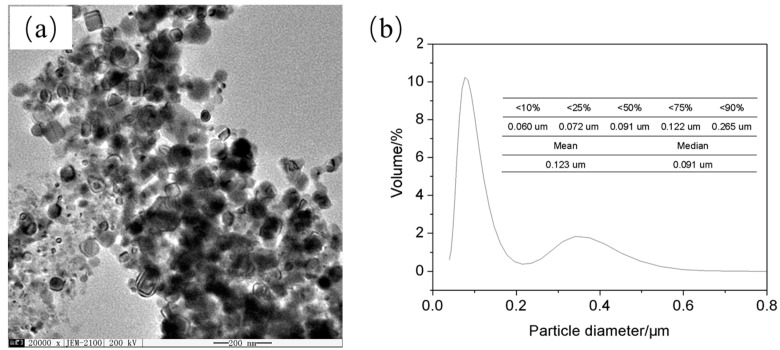
TEM image (**a**) and particle size distribution (**b**) of the product [96].

We synthesized ZrB_2_ nano powders successfully in an RF thermal plasma under atmospheric pressure, in which ZrCl_4_, B, and Mg as raw materials were mixed as the green mixture. The metallothermic reaction was ignited by the high temperature flame when the green mixture was carried by H_2_ into thermal plasma, and then propagated with the exothermic reaction enthalpies and the external energy supplied by plasma flame. Scheme 5 shows the process flow chart for plasma synthesis of ZrB_2_ nano powders. Figure 11 shows the TEM image and particle size distribution of the product [95,96].

It can be seen that the particle size of the synthesized ZrB_2_ powders is about 100 nm and the surface area of the nanopowders is 30.75 m^2^/g. It was a continuous production process and the production rate in our laboratory was about 300 g/h. This plasma-enhanced exothermic reaction was further used for the synthesis of ultrafine ZrB_2_-ZrC composite powders using ZrCl_4_, B, Mg, and CH_4_ as raw materials. Detailed information was reported in the literature [97].

## 6. Perspective Conclusions

In recent years, scientists and technological workers have carried out a lot of in-depth research around three key problems, including the preparation technology of high-purity, ultra-fine, and homogeneous ceramic powders, the molding process of low-cost complex shape ceramic parts, and advanced sintering theory and sintering technology, and have made great breakthroughs in development and application. High-purity, ultra-fine, and homogeneous powders are the basis for the preparation of UHTCs and their composites. Traditional synthesis methods have encountered various difficulties in meeting the needs of rapid development. Therefore, new synthesis methods have broken through the traditional classification of the solid-phase method, the liquid-phase method, and the gas-phase method, and there is a trend of integration of them. The present review covers most important methods used in ZrB_2_ nanopowder synthesis, focusing on the solid-phase synthesis and its improved process, including modified self-propagating high-temperature synthesis, the solution-derived precursor method, and plasma-enhanced exothermic reactions.

In the future, ultra-high temperature ceramics will still develop towards exploring extreme parameters, for which it is often necessary to change the original properties of basic materials, and the most effective methods to change the properties include particle refinement and recombination in the ultra-fine state. Furthermore, physical and chemical problems in powder preparation have a strong correlation with scientific problems in the molding process and thermodynamics and kinetics in the firing process, and solution of these basic problems is the basic guarantee for obtaining material reliability and stability. Therefore, the integration of various synthesis methods, the combination of different material components, and the connection between synthesis and its subsequent application process are the trends of development in the future.

## Figures and Tables

**Table 1:** Crystallite size of different synthesized powder samples [62].

NaCl (wt.%)	Crystal Size (nm)
0	25
5	20
10	18
15	16
20	13

**Table 2:** Comparison list between the characteristics of plasma process and the properties of ZrB_2_ powders.

Characteristics of Plasma Process	Properties of ZrB_2_ Powders
High temperature	High temperature
Rapid cooling	Nano size
No electrode pollution	High purity
Controllable atmosphere	Non-oxide
